# Supplementary material for: Does a Specific Sequential Combination of Antiseptic Solutions for Chemical Debridement in Periprosthetic Joint Infection Improve Outcomes vs. Solution Alone? An In Vivo Study
Source: Antibiotics (Basel). 2024 Dec 17;13(12):1225. doi: 10.3390/antibiotics13121225 (PMC11727590; doi:10.3390/antibiotics13121225)

SUPPLEMENTAL MATERIAL

Figure S1: Prevalence of limping and piloerection in mice following surgery.

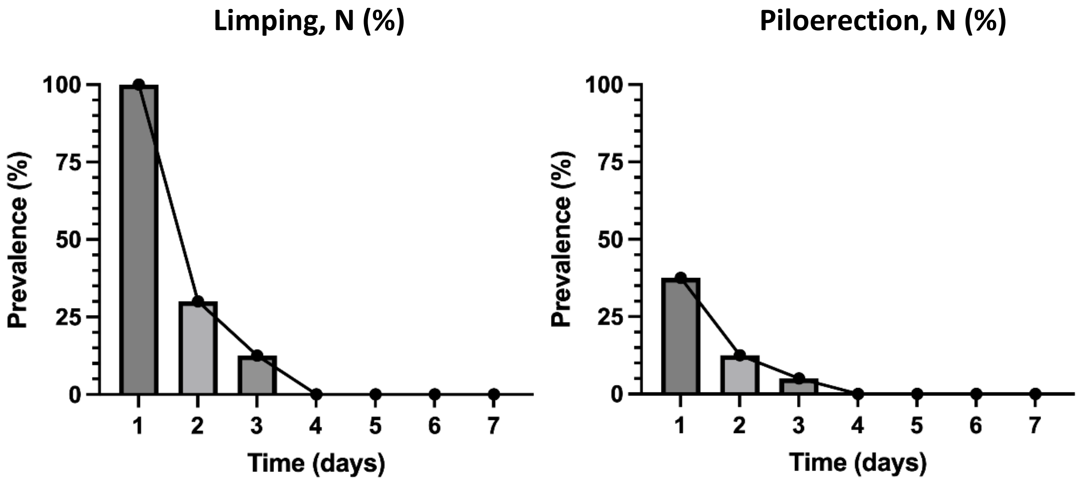

Supplement: Supplementary file 1 [file antibiotics-13-01225-s001.zip › antibiotics-3315455-supplementary.pdf]
